# Protective Behavior Survey, West Nile Virus, British Columbia

**DOI:** 10.3201/eid1008.031053

**Published:** 2004-08

**Authors:** Michael Aquino, Murray Fyfe, Laura MacDougall, Valencia Remple

**Affiliations:** * University of Toronto, Toronto, Canada;; † British Columbia Centre for Disease Control and Prevention, Vancouver, Canada

**Keywords:** West Nile virus, knowledge, attitude, British Columbia, behavior, DEET, mosquito, vector, dispatch

## Abstract

We investigated personal protective behaviors against West Nile virus infection. Barriers to adopting these behaviors were identified, including the perception that DEET (N,N-diethyl-m-toluamide and related compounds) is a health and environmental hazard. Televised public health messages and knowing that family or friends practiced protective behaviors were important cues to action.

Personal protective behaviors are the primary means of preventing human illness from West Nile virus (WNV) infection (1). To plan effective WNV prevention and control programs, we must know the factors that influence adopting protective behaviors (2). The health belief model is a theoretical framework that has investigated health behaviors related to infectious diseases, including tuberculosis, HIV, influenza, and measles (3–8). The major tenet of this model is that persons will take action to ward off an illness if they believe that they are susceptible, the illness has serious consequences, the course of action is beneficial, or the anticipated benefits of action outweigh the costs (9). To investigate the determinants of engaging in WNV protective behaviors in British Columbia (B.C.), we developed a questionnaire using the health belief model as a framework.

## The Study

Participants were randomly selected from a systematic random sample of B.C. residential telephone records. Telephone interviews were conducted from July 2 through August 18, 2003. The study concluded after 309 interviews were completed, a predetermined endpoint based on allowable resources.

A questionnaire was designed specifically for this study. We measured the frequency (1 = never to 5 = always) with which participants said they practiced protective behaviors (applied mosquito repellent, eliminated standing water, and avoided mosquitoes). The following predictor items measured concepts of the health belief model: knowledge (mode of transmission, risk groups), susceptibility to illness, severity of illness, barriers to action (safety concerns, cost), benefits of action, and cues to action (behavior of relatives, sources of information). Response options for predictors were measured on a 5-point Likert scale and signified the respondent's agreement with a statement (1 = strongly disagree to 5 = strongly agree) or belief that an event would occur (1 = not at all likely to 5 = very likely). Response options for predictors measured with multiple-choice questions were scored as correct or incorrect. The subscore for a concept was calculated by averaging the scores obtained from questions specific to that concept. The subscore for the knowledge concept was calculated by counting the number of correct responses. We ascertained the participant's sex, age, ethnicity, education, and income. Participants were also asked to report the level of mosquito activity near their residence (low, medium, high).

A response rate of 64.6% (307 of 477) was calculated by dividing the number of completed surveys by the number of persons contacted. Respondents were more likely to be women, older, more educated, and in a higher income bracket than the general population.

Most respondents said they obtained information about WNV by watching television (n = 180, 62.9%). While almost all (n = 285, 98.6%) were aware that WNV was transmitted by mosquitoes, 57.9% (n = 159) were aware that adults >50 years of age are at greatest risk for serious illness. Of those unaware of this fact, 52.0% (n = 63) were >50 years of age.

At least occasional practice of the following specific protective behaviors was reported: 197 (68.2%) removed standing water, 168 (58.1%) practiced mosquito avoidance behavior, and 162 (56.0%) used DEET-based mosquito repellents (Figure). When asked if information about WNV had influenced them to remove standing water, agreement or strong agreement was reported by 213 (73.7%) respondents, 147 (50.9%) for using DEET (N,N-diethyl-m-toluamide and related compounds)-based mosquito repellent, and 110 (38.1%) for avoiding mosquitos.

**Figure:**
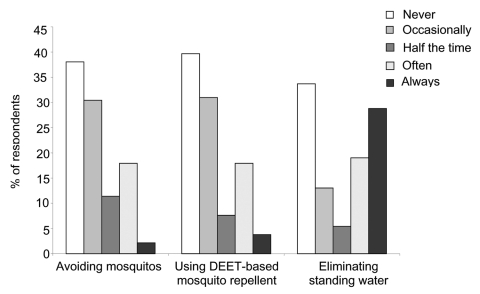
Reported frequency of personal protective behavior practice. DEET, N,N-diethyl-m-toluamide and related compounds.

The most prominent barriers to practicing protective behaviors were perception that DEET is a health and environmental hazard, the time required to remove standing water, and participating in outdoor leisure activities during peak mosquito hours. Approximately half (n = 113, 45.9%) of respondents claiming to have spent time outdoors during peak mosquito hours did so to participate in leisure activities (walking, playing with kids, gardening). More than one third (n = 101, 35.1%) agreed or strongly agreed that removal of standing water was time consuming. More than one third (n = 101, 35.1%) believed DEET is hazardous to the environment, and more than one quarter (n = 78, 27.1%) disagreed or strongly disagreed that it is safe for human use.

The proportional odds ordinal regression model was fit to model the frequency at which persons reported avoiding mosquitoes, applying DEET, and removing standing water (Table). Predictors that were investigated included the composite scores for each of the health belief model concepts, demographic variables (sex, age, area code, education, income, ethnicity), and residential mosquito activity level.

**Table:** Results of modeling reported frequency of personal protective behavior with proportional odds ordinal regression^a^

Outcome modeled	Significant predictor variables	β	OR (95% CI)	p value
Practicing mosquito avoidance behavior	Perceived barriers to action	–0.77	0.46 (0.35–0.62)	< 0.01
	Cues to action	1.08	2.96 (2.32–3.77)	< 0.01
	Perceived susceptibility	0.48	1.61 (1.22–2.12)	< 0.01
Reported frequency of using DEET-based mosquito repellent	Perceived barriers to action	–0.70	0.50 (0.31–0.79)	< 0.01
	Cues to action	1.19	3.30 (2.49–4.38)	< 0.01
Reported frequency of eliminating standing water	Perceived barriers to action	–1.25	0.29 (0.20–0.42)	< 0.01
	Cues to action	1.27	3.56 (2.49–5.09)	< 0.01

^a^OR, odds ratio; CI, confidence interval; DEET, N,N-diethyl-m-toluamide and related compounds.

Barriers to action and cues to action were important predictors in each of the three models. In addition, perceived susceptibility was significant in modeling the frequency of practicing avoidance of mosquitoes (p < 0.01) and had an associated odds ratio of 1.61 (confidence interval 1.22–2.12). All other investigated predictors were not significant in multivariate models at the α = 0.05 level.

## Conclusions

Before the study, the British Columbia Centre for Disease Control and Prevention issued three formal press releases and provided 110 interviews with local or provincial media outlets about WNV and associated protective behaviors. Most respondents in our study cited television as their main source of information, which demonstrates the ability of public health messaging to reach audiences through broadcast media. This finding is consistent with findings from other studies (10) and underscores the important role that this medium plays in educating the public (11).

The proportion of respondents who said they used DEET-based mosquito repellent or practiced mosquito avoidance behavior was comparable to the proportion found in similar studies conducted in Connecticut (2,10). Eliminating standing water was not specifically investigated in these studies. A national U.S. study reported smaller proportions of respondents who said that they avoided the outdoors during dawn or dusk (24%), used DEET-based mosquito repellent (31%), and eliminated standing water (31%) (12). Differences may be the result of the varying levels of WNV activity throughout the United States.

Regular systematic evaluations of the knowledge, attitudes, and behaviors of the public are needed to ensure the effectiveness of public health messages (2). By using the health belief model as a theoretical framework, we were able to identify barriers to the practice of protective behaviors. The fear that DEET-based mosquito repellents are hazardous to human health and the environment is a barrier of particular concern. These repellents are a mainstay for the personal prevention of WNV (11) and were demonstrated to be an important protective behavior option, given the participation of many respondents in outdoor activities during peak mosquito hours. Instructions for the safe use of DEET are outlined in the literature (13) and should be conveyed to address public fears. Literature on the effects of DEET on the environment is limited. DEET does not readily degrade by hydrolysis at environmental pHs (14) and has been identified as a ubiquitous pollutant in aquatic ecosystems, but the effect of this is unknown (15).

A deficiency was also observed in the proportion of respondents who were aware that persons >50 years of age were at greatest risk for serious illness from WNV. More than half of those unaware were >50 years of age. Making perceptions of susceptibility and severity in this population more consistent with the actual susceptibility and severity could help to influence the adoption of WNV protective behaviors (9).

Our study supports the ability of public health education campaigns to influence the practice of WNV protective behaviors. Specifically, we found that most respondents reported that information about WNV influenced them to engage in protective behaviors, and cues to action significantly increased the odds that respondents practiced protective behaviors more frequently. Together, these findings suggest the potential for public health messages that endorse WNV protective behaviors to have a "snowball effect"; public health education can influence persons to practice protective behaviors, and these persons can influence friends and family to do the same.

A number of limitations were associated with our study. First, we depended on self-reporting to measure frequency of practicing WNV protective behaviors, and no effort was made to validate the participants' responses. If participants attempted to please interviewers, the frequency of protective behavior practice may have been overestimated. Second, administering the interview by telephone excluded persons who did not have telephones or only had cellular phones, which may have contributed to the observed demographic differences between our study population and the general population. Consequently, findings may not be generalizable outside the study population. Third, information on nonrespondents was not obtained, and differences between them and the study population could not be ascertained. Thus, the effects of this bias could not be determined. Despite these limitations, this study will help public health officials achieve the goal of promoting WNV protective behaviors and reducing the risk for infection.
