# Correction, Vol. 10, No. 8

**DOI:** 10.3201/eid1009.C31009

**Published:** 2004-09

**Keywords:** errata, erratum, correction

For the article by Michael Aquino et al., p. 1499, the correct title is “Protective Behavior Survey, West Nile Virus, British Columbia.”

The corrected article appears online at http://wwwnc.cdc.gov/eid/article/10/8/03-1053_article.htm.

We regret any confusion this error may have caused.

